# California State Dental Association

**Published:** 1873-07

**Authors:** H. J. Plomteaux


### Proceedings of Societies.

### CALIFORNIA STATE DENTAL ASSOCIATION.

Pursuant to adjournment, the fourth annual session convened in St. Andrews Hall, Young Mens, Christian Association Building, San Francisco, May 20th. 1873, and was called to order at 10 o'clock A. M. by the President, Wm. Dutch D. D. S.

Prayer by Rev. Dr. Cox, of San Francisco.

Officers all present during the entire session, except Vice President S. M. Harris, who was absent on account of illness.

### NEW MEMBERS.

During the session which continued four days, the following named gentlemen were admitted to active membership; J. W. Winter, Alex. Warner D. D. S., C. W. Richards, D. J. Nis, W. H. Stanley, J. B. Beers, F. H. Furgerson D. D. S., C. H. Fuller, and A. B. Wood, of San Francisco; G. C. Hoadley, of San Jose; F. H. Hubbard, M. D. of Sacramento; J. J. Dyar, of Santa Cruz; D. M. Powers, of Oakland, and A. Cornwall, of Virginia City Nevada.

### HONORARY MEMBERS.

Dr. O. D. Munson, E. S. Belden, M. D., of San Francisco and B. S. Codman, of Boston Mass.

### ESSAYS, were read as follows;

Requisites of the Dentist, - - - - by W. J. Prather.
Circulation of the Blood, - - - - „ H. Austin.
The Mouth, its structure and Function, „ R. Cutlar.
Operative Dentistry, - - - - - - „ J. M. Myers.
Causes and Treatment of Offensive breath, by Max Sichel.
Dental Ethics, - - - - - - - - - - by F. E. Bunnell.

### REPORTS OF STANDING COMMITTEES.

Reports were received from Drs. Menefee, Bush and Mc Cowen of Com., on Mechanical Dentistry, and Dennis on Operative Dentistry. Also from S. E. Knowles, on Dental Pathology and Surgery.

SPECIAL COMMITTEES.

The Legislative Committee reccommended a bill similar to the Ohio law, giving members of the profession, non-residents of the state, two years to prepare for an examination.

Report adopted, and Com. instructed to use all legitimate means to secure its passage at next session of the Legislature.

The revision Com. reported a revised Constitution, By Laws, Code of Ethics, etc., which were adopted. The Code of Ethics is the same as that of the American Dental Association.

The Barnum Testimonial Committee, accompanied their report with a magnificent testimonial, which will be presented to Dr. Barnum at the next session of the American Dental Association, therefore, I refrain from giving a description of it here. Although, as remarked by the Com., it is a paltry gift to one who has proven himself a public benefactor, by bestowing such a noble *free gift* upon the profession, when he had a fortune within his reach, had he patented his invention. Paltry though it be, it is one that he will be proud of, and it will reflect credit upon our Association.

DENTAL COLLEGE.

President Dutch in his annual address, said : "It is evident to us all, that we need on the Pacific coast, a school of instruction, for those who would qualify themselves for the practice of Dentistry. A school where the science and the art, the theory and the practice of dentistry may be acquired, and the power of publishing to the world as an authority to be respected, the proficiency of those who have been thoroughly taught, as by the giving of diplomas." He also recommended the selection of *eight* from our numbers, who might be deemed the best qualified, and "who were willing to take upon themselves such a responsibility, requesting them to organize from among themselves, as far as they can, a Dental College, affiliating with the University of California or some other city organization, for the purpose of conferring the degrees of D. D. S. Or failing in such satisfactory affiliation, then asking the Legislature to confer the power on the college itself, and that you give such an organization all the support you can individually and collectively."

Prof. Gillman, Professor of the University of California, having been invited, addressed the Association on the above subject. He spoke of the University as an "institution of learning, established by the State on foundations which were laid by private devotions in the days of Argonauts, who believed in the wisdom which was better than gold. It has been endowed by the National Government with munificent land grants, and received from the State generous donations and a comprehensive charter. It is a University of the nineteenth century, open without money and without price to students of every state and country, designed to promote the efficiency of every branch of liberal culture."

He would be glad if in the organization of a Dental School in connection with the University, they could rely upon the co-operation of the California State Dental Association. All the authorities of the University would rejoice at suggestions and help and counsel from members of this society.

The Professor's address was received with applause, and he concluded by saying that he had "always dreaded meeting a single gentleman of the profession, and dreaded much more coming into the hands of thirty or forty," but hoped from the reception given him, that he would be able to report that we were united, and would be found cooperating with the University in the interests of science and humanity.

A committee of five was appointed to confer with the Board of Regents, relative to the matter and further consideration of it made the special order for 10:30 o'clock A. M., next day when the committee reported the following resolutions, which were unanimously adopted :

*Resolved*, that this Association receives with unqualified satisfaction the assurances that our most cherished hopes are to be realized through the wisdom and liberality of the Board of Regents, of the University of California, in their proposed establishment of a dental college.

*Resolved*, that we approve of and recommend the following curriculum of instruction in the College : 1. General Anatomy and Surgery ; 2. Physiology ; 3. Materia Medica and Therapeutics : 4. Chemistry and Toxicology ; 5. Special Anatomy and Surgery ; 6. Operative Dentistry and Pathology ; 7. Dental Prosthesis and Metallurgy ; 8. Institutes of Dental

Science ; 9. Demonstrator of Operative Dentistry ; 10. Demonstrator of Dental Prosthesis ; 11. Course of Clinical Lectures ; 12. Board of Examiners.

*Resolved*, that the Association endorse and recommend the following named eight gentlemen as proper persons to fill the special chairs above designated : Drs. Wm. Dutch, S. W. Dennis, C. C. Knowles, Wm. J Younger, H. E. Knox, S. E. Knowles, H. Austin and J. J. Menefee.

*Resolved*, that an Advisory Committee of five be appointed, and empowered to act with the Advisory Committee of the Board of Regents, in carrying into effect all necessary details of the organization.

The President appointed Drs. Cutler, Lundborg, Birge, Bunwell and Cogswell to act as Advisory Committee.

CLINICS

Were given during the session by Drs. Dutch, Dennis, Knowles, Knox and Younger.

DISCUSSIONS.

The several reports and essays were thoroughly discussed, and as soon as published each dental journal will be furnished a copy so that they can select therefrom any matter they may deem new or important to their readers, and the profession generally.

OFFICERS.

Our officers for the ensuing year are :

President—Dr. C. C. Knowles, San Francisco ; Vice President—J. J. Menefee, San Jose ; Secretary—H. J. Plomteaux, Woodland ; Corresponding Secretary—H. E. Knox, San Francisco ; Treasurer—H. Austin, San Francisco ; Librarian—J. J. Birge.

STANDING COMMITTEES

Were appointed on Dental Pathology and Surgery, Dental Therapeutics, Dental Chemistry, Operative Dentistry, Mechanical Dentistry, Histology and Microscopy, Dental Literature and Education, Publication and Executive.

THESES.

The President made the following assignments :

An inquiry into the Causes and Treatment of Degeneracy of Children's Teeth—Dr. D. M. Hughes.

The Secretions of the Mouth, Considered in their Normal and Abnormal Characteristics—T. H. Furgerson.

Pregnancy as Effecting the Teeth—H. Cornwall.

The Interelations of Dentistry and Medicine—J. A. W. Lundborg.

Extraction of Teeth—F. M. Shields.

Dental Education—H. J. Plomteaux.

THE NEXT ANNUAL SESSION

Will be held in San Francisco, commencing on the second Tuesday in June, 1874, at 10 o'clock A. M.

Resolutions appropriate to the memory of Dr. E. O. Belle, deceased since our last annual session, were reported and adopted by standing vote.

Mr. F. P. Forbes, of San Francisco, presented the Association, for use of the President, an elegant marble slab and gavel, which was received with applause; and a vote of thanks extended the donor.

The able and efficient administration of President Dutch was highly endorsed by the Association.

The Session lasted four days, and, with short intermissions, was at work from 10 o'clock A. M., until 10 o'clock P. M.

After adjournment we proceeded to Dr. Martin's Restaurant, and partook of an excellent collation, given us by the San Francisco Dental Association. H. J. PLOMTEAUX.

*Secretary.*

